# Addressing global disparities in neurosurgical workforce and access to care

**DOI:** 10.1186/s41016-025-00419-1

**Published:** 2025-11-20

**Authors:** Ehsanullah Alokozay, Ehtisham Haider, Neha Waseem, Najibullah Alokozay

**Affiliations:** 1 Faculty of Medicine, Kandahar University, Kandahar, 3801 Afghanistan; 2 Faisalabad Medical University, Faisalabad, Pakistan; 3 FMH College of Medicine and Dentistry, Lahore, Pakistan; 4 Kabul University of Medical Science “Abu Ali Ibn Sina”, Kabul, Afghanistan

## Abstract

Neurosurgical care remains inaccessible to over two-thirds of the global population, with the greatest burden falling on low- and middle-income countries (LMICs). Neurological disorders contribute to nearly 9 million deaths annually, while an estimated 22.6 million new cases require neurosurgical attention each year. Workforce shortages, particularly in Africa and Southeast Asia, exacerbate this crisis, with many countries falling below the minimum target of 0.5 neurosurgeons per 100,000 population. Beyond workforce deficits, systemic barriers, including limited access to training, mentorship, funding, and equitable career advancement, compound disparities and hinder long-term retention. The Boston Declaration 2025 and the World Health Organization (WHO) Intersectoral Global Action Plan emphasize the integration of neurosurgical services into national surgical, obstetric, and anesthesia plans, alongside investment in mentorship, inclusivity, and institutional support. Telemedicine has shown promise in expanding access through remote consultations, teaching, and follow-up care, yet infrastructure and policy challenges persist. This correspondence focuses on addressing global inequities in neurosurgery, which requires multipronged strategies: workforce expansion, digital health adoption, systemic reforms, and embedding neurosurgical care into broader health frameworks. Sustainable progress will depend on consistent investment, evidence-driven policies, and global collaboration to ensure equitable access to neurosurgical care worldwide.

Dear Editor,

With over two-thirds of the global population lacking quality neurosurgical services, particularly in low- and middle-income countries (LMICs), there is an urgent need for sustained advocacy and policy reform. The Boston Declaration 2025 emphasizes the importance of developing long-term capacity, aligning with global strategies to address disparities in access to care. This is in contrast to earlier calls to action, such as that by Lartigue et al., which primarily emphasized the integration of neurosurgery into health systems, and the Boston Declaration 2023 provides a more targeted approach [1]. Neurological conditions account for 9 million deaths annually, with most of this burden concentrated in LMICs [2, 3].

Global neurosurgical needs are increasingly well defined. An estimated 22.6 million cases are reported annually, of which 13.8 million require surgical intervention, largely in LMICs. The current deficit of about 23,300 neurosurgeons, particularly in Africa and Southeast Asia, underscores the mismatch between demand and available expertise [4]. The 2024 global workforce mapping update reported a pooled density of 0.93 per 100,000 and a median country density of 0.44 per 100,000, with many LMICs far below the minimum workforce target of 0.5 per 100,000 by 2030. If current trends persist, more than half of LMICs are projected to miss this benchmark [5]. This data has been summarized in Table 1.

**Table 1:** Summary of neurosurgical workforce estimates

Parameter	Estimate
Global annual neurosurgical cases	22.6 million
Global annual neurosurgical cases requiring intervention	13.8 million
Neurosurgeon deficit in Africa and Southeast Asia	23,300 neurosurgeons
Pooled global neurosurgeon density	0.93 per 100,000
Median country neurosurgeon density	0.44 per 100,000
Workforce target by 2030	0.5 per 100,000

In addition to the inequities that are highlighted at a global level, there are sharp differences between and within regions. In certain African countries, a single neurosurgeon may be responsible for millions of patients. Most neurosurgeons are based in major urban centers, which leave people in rural areas with little or no reliable access to timely emergency neurosurgical care. This results in preventable delays, worse outcomes, and an extra financial burden for families who need to travel far for treatment [4, 5]. Workforce expansion alone is insufficient without addressing systemic barriers to training and retention. The World Federation of Neurosurgical Societies (WFNS) Young Neurosurgeons survey, which included 500 early-career neurosurgeons worldwide, identified limited access to advanced training (56.1%), insufficient funding (50.6%), lack of mentorship (48.2%), and significant work-life imbalance (87.1%) as common challenges, with disproportionate effects in LMICs. Shitsama et al. further underscored the impact of impaired work-life balance on the ongoing scarcity of the neurosurgical workforce [6]. These challenges were further complemented in a regional study from Africa that emphasized the lack of availability of training and research opportunities for neurosurgeons in LMICs [7]. These barriers hinder career development and perpetuate workforce shortages. Addressing them requires structured mentorship, equitable funding, and supportive policy interventions [8].

In addition to the disparities observed in training, funding, mentorship, and work-life balance, concerns about burnout and retention remain significant. Almost 90% of respondents reported difficulty balancing work and personal life, suggesting that long-term participation in the field may be difficult even for trained neurosurgeons. Continued disparities in gender and race further restrict the pipeline of new talent. Establishing mentorship programs, expanding leadership opportunities, and enhancing institutional support are crucial to developing a workforce that can thrive and remain inclusive [8].

To address the global and regional disparities in the neurosurgical workforce, encouraging progress has been demonstrated through targeted initiatives. The Boston Declaration 2025 reported global pledges and commitments to expand training programs, foster public–private partnerships, and strengthen advocacy at the policy level [9]. While the expansion of the workforce is still being measured, the declaration has brought together global neurosurgery initiatives and drawn attention to inequities in access.

One of the key contributions of the declaration is the way it connects neurosurgery with broader global health efforts. Tying it to national surgical, obstetric, and anesthesia plans (NSOAPs) allows neurosurgical care to be part of long-term health planning rather than in isolation. It further highlights the need for accountability and clear metrics, acknowledging that promises by themselves are not enough to close capacity gaps [9]. Telemedicine is increasingly used as a complementary approach. In neurosurgery, applications range from online consultations to postoperative care and remote training, making it especially important in resource-limited regions of LMICs. Tenkorang et al. note benefits such as reduced travel burdens and improved results while also pointing out ongoing obstacles like connectivity issues, policy gaps, and workforce capacity [10].

Building on the benefits of telemedicine as shared by Tenkorang et al., the provision of remote intraoperative consultations, online tumor boards, and virtual teaching sessions has made specialized knowledge more widely available. Patients also benefit from remote follow-up, which cuts down on the expense and difficulty of frequent travel. Yet, serious obstacles remain, including limited Internet access, unclear licensing rules, and weak reimbursement systems [10].

Findings from the Global Burden of Disease Study indicate that age-standardized neurological disability-adjusted life years (DALY) rates have stabilized or declined in certain areas. However, LMICs continue to face a steady increase in total DALYs, mainly due to demographic changes. With aging populations, an increase in high trauma cases among the young, and a growing burden of stroke and degenerative spine disease among older adults, these demographic changes highlight an incremental burden on the already scarce neurosurgical workforce, underscoring the need for surgical capacity to grow in step with population changes [3].

Young neurosurgeons have pointed out that the challenges go beyond training limitations, drawing attention to systemic inequities. The WFNS survey documented persistent disparities in career advancement, with 40.5% of participants citing gender bias and 22.4% reporting racial or ethnic bias [8]. These results highlight the need for workforce planning that also focuses on creating inclusive professional spaces. Promoting equity is essential to both long-term workforce sustainability and to better reflect the communities served.

Addressing workforce gaps must go hand in hand with wider system strategies. Considering the limited availability of resources in LMICs, task sharing can play a crucial role. It can be achieved with the involvement of medical practitioners, who can play a role in the initial evaluation of patients, thereby minimizing the burden on the neurosurgical workforce. Community health workers can contribute by raising awareness and promoting screening to enable early diagnosis and prompt treatment, thereby reducing the healthcare burden. The importance of task sharing has been further highlighted in the WHO Intersectoral Global Action Plan on epilepsy and other neurological disorders (2022–2031), which emphasizes the benefits of integrating neurological care into primary health systems. Where neurosurgeons are unavailable, trained nonspecialists who can provide emergency stabilization play a vital role in saving lives. Coupled with training investments, such measures create stronger systems that can function beyond the availability of scarce specialists [2].

This strategy can facilitate both in the short term and the long term. Emergency neurosurgical training can be provided to health care professionals at a local level, which can then be expanded on a regional level with government partnership. Prospectively, in accordance with the WHO Intersectoral Global Action Plan on epilepsy and other neurological disorders (2022–2031), the incorporation of neurosurgical care into the national health system would become more feasible with the increased availability of trained professionals as a result of earlier training initiatives. Additionally, the limited availability of resources in LMICs hinders their participation in simulation-based research. This contrasts significantly with high-income settings where simulation-based training plays a crucial role, further adding to the global disparities in the neurosurgical workforce. This focuses on the importance of global investment and collaboration [11].

Closing gaps in global neurosurgery requires joint efforts to grow the workforce, remove systemic obstacles, adopt digital solutions, and integrate frameworks such as the Boston Declaration into policy. The complementary priorities outlined in the Boston Declaration and the WHO Intersectoral Global Action Plan offer a road map for advancing these goals. Universal access will rely on consistent investment, evidence-based approaches, and sustained global collaboration.

## Data Availability

No datasets were generated or analysed during the current study.

## Abbreviations

LMICs: Lower- and middle-income countries
WHO: World Health Organization
DALY: Disability-adjusted life years
WFNS: World Federation of Neurosurgical Societies
